# Secondary Radial Nerve Palsy after Minimally Invasive Plate Osteosynthesis of a Distal Humeral Shaft Fracture

**DOI:** 10.1155/2015/241968

**Published:** 2015-10-08

**Authors:** Ursina Bichsel, Richard Walter Nyffeler

**Affiliations:** ^1^Clinic of Cardiovascular Surgery, Inselspital, Bern University Hospital, University of Bern, 3010 Bern, Switzerland; ^2^Sonnenhof Orthopaedic Centre, Orthopädie Sonnenhof, Buchserstrasse 30, 3006 Bern, Switzerland

## Abstract

Minimally invasive plate osteosynthesis is a widely used procedure for the treatment of fractures of the femur and the tibia. For a short time it is also used for the treatment of humeral shaft fractures. Among other advantages, the ambassadors of this technique emphasize the lower risk of nerve injuries when compared to open reduction and internal fixation. We report the case of secondary radial nerve palsy caused by percutaneous fixation of a plate above the antecubital fold. The nerve did not recover and the patient needed a tendon transfer to regain active extension of the fingers. This case points to the importance of adequate exposure of the bone and plate if a humeral shaft fracture extends far distally.

## 1. Introduction

Minimally invasive plate osteosynthesis is an upcoming procedure for the treatment of unstable fractures of the upper extremities. The technique consists of insertion and fixation of a plate through separate small incisions. It is thought to preserve the fracture hematoma and the vascular supply to the fracture fragments and to cause less pain and scarring. It has been used for the treatment of proximal [1, 2], middle [3], and distal [4, 5] humeral shaft fractures. All authors reported a high rate of bony healing and a low rate of complications, including nerve injuries.

The purpose of this case report was to recognize the course of the radial nerve and to point out the danger of an iatrogenic lesion of this nerve in the distal part of the humerus.

## 2. Case Report

**Figure 1:**
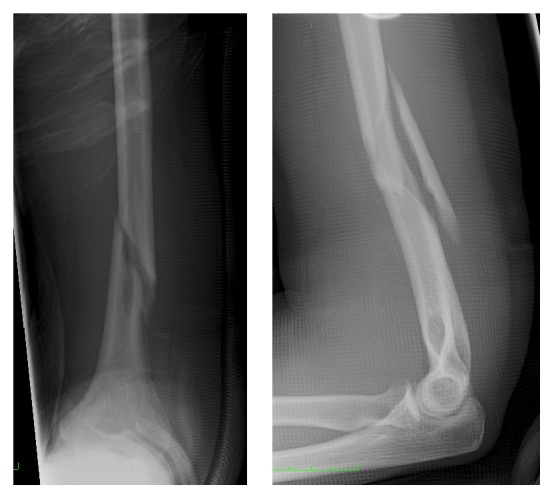
Preoperative anteroposterior and lateral radiographs of the slightly displaced distal humeral shaft fracture with a long wedge fragment.

**Figure 2:**
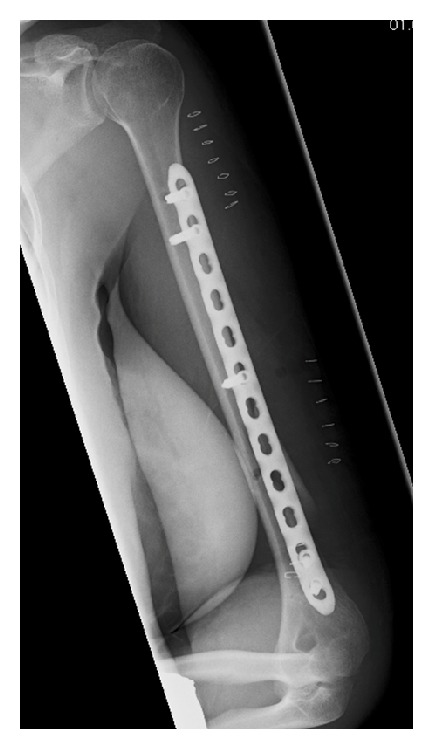
Postoperative radiograph shows the minimally invasive plate osteosynthesis and the skin staples.

**Figure 3:**
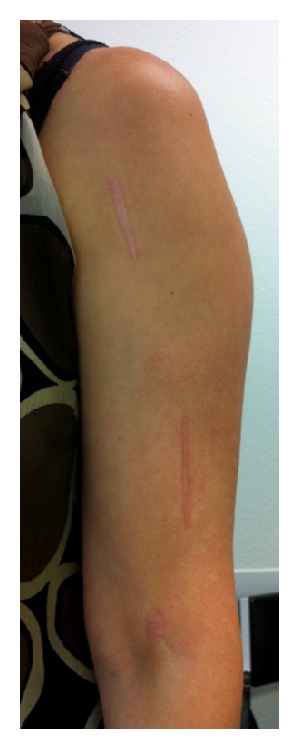
Photograph of the left arm of the patient shows the three scars of the minimally invasive operation technique. Distally a stab incision was made above the antecubital fold.

A 39-year-old woman, who fell during sledging, sustained a slightly displaced long spiral fracture of the distal third of the humeral shaft with a wedge fragment on the posteromedial side (AO classification 12-B1) (Figure 1). The sensibility and motor function of the hand were normal. The surgeon on call recommended the operative treatment and performed a minimally invasive plate osteosynthesis on the next day. A short incision was made on the lateral side of the arm, at the level of the fracture. The radial nerve was carefully exposed and protected. The main bone fragments were then reduced and a 12-hole plate was inserted through a small anterolateral incision more proximally. The plate was pushed distally, between the musculature and the periosteum. It was fixed to the bone with two screws proximally, a screw in the middle of the shaft and two screws distally. The distal screws were inserted percutaneously through a stab incision, just above the antecubital fold (Figures 2 and 3). The postoperative radiographs showed a correct fracture alignment, and the clinical examination revealed a drop hand and a loss of sensibility. Electromyography confirmed complete radial nerve palsy. The surgeon did not recognize an intraoperative injury to the nerve and decided to wait and see. The patient's wrist and fingers were stabilized with a radial substitute splint. Physical and occupational therapy were started immediately to prevent joint contractures. After three months the patient could slightly raise the hand. Full extension of the wrist was possible after 8 months. Active extension of the fingers, however, remained impossible. A tendon transfer was therefore made 13 months after the initial surgery. This procedure was successful and the patient could resume her activity as secretary. She was referred to our institution for an expert opinion. She consented that data concerning her case would be submitted for publication.

## 3. Discussion

Minimally invasive plate osteosynthesis is a safe procedure for the treatment of fractures of the lower extremities. The plates are placed on the lateral side of the distal femur and on the medial side of the tibia. In these areas there are only cutaneous nerves but no motor branches that could be damaged during insertion or fixation of the plates.

The situation is different in the upper extremity. The soft tissue envelope is thicker and there are two important motor nerves that cross the operating field: the axillary nerve proximally and the radial nerve in the middle of the shaft and distally. The axillary nerve winds around the humerus, from posterior to anterior, on the deep surface of the deltoid muscle, and about 7 cm below the acromion. It can be damaged if a deltoid split is extended more distally. A lesion of this nerve results in an atrophy of the muscle belly anterior to the split and a weakness for active flexion and elevation of the arm.

The radial nerve crosses the posterior aspect of the humerus in the spiral groove, between the lateral and medial head of the triceps. It penetrates the lateral intermuscular septum and moves anteriorly and inferiorly between the brachialis and brachioradialis muscles. During its course it gives motor branches to the above-mentioned muscles, as well as to the extensor carpi radialis longus and brevis. In the cubital fossa it divides into the superficial radial nerve and the deep motor branch, which innervates the extensors of the fingers.

**Figure 4:**
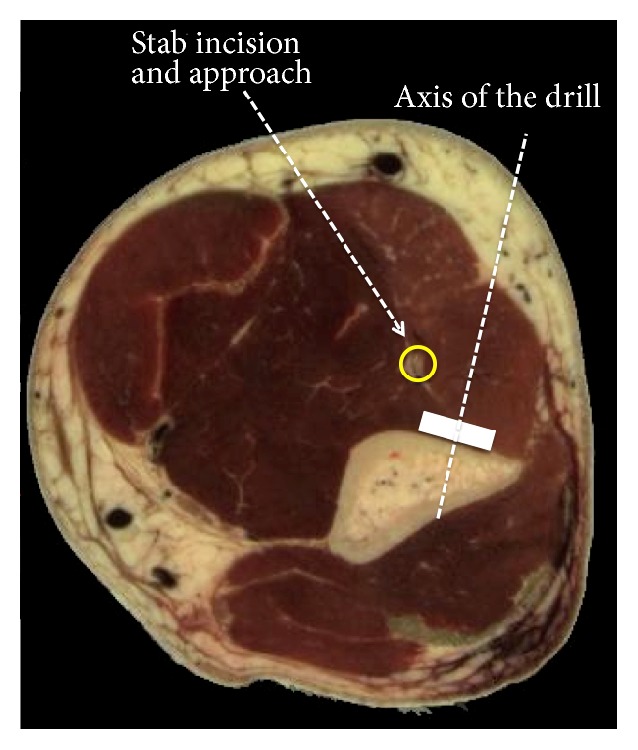
Cross section of the left arm of an adult woman (visible woman server, EPFL, Switzerland) demonstrates the relationship between the stab incision above the antecubital fold, the radial nerve, and the plate on the anterolateral aspect of the distal humerus. The radial nerve (yellow circle) is embedded between the brachialis and brachioradialis muscles, in a line connecting the stab incision above the antecubital fold and the plate.

Most radial nerve lesions occur during the trauma and already exist before the surgical intervention. Secondary nerve lesions may occur during preparation of the patient and disinfection of the unprotected arm or during the surgical procedure itself. The nerve can be entrapped between the bone fragments or between the bone and the plate; it can sustain a traction lesion or be damaged by a forceps, a knife, or a drill bit. In the present case the nerve was damaged distally, just above the antecubital fold. At this level the nerve is in a line connecting the skin incision and the distal holes of the plate (Figure 4). In order to make the drill holes perpendicular to the bony surface, the surgeon needed to align the drill bit by pulling the soft tissues laterally. The radial nerve was therefore at risk during insertion (direct damage) and reorientation (traction injury) of the drill bit and the screws. The fact that active extension of the fingers did not recover indicates that the nerve was rather cut than stretched.

Baumann et al. described 3 cases of radial nerve disruption following percutaneous placement of a hinged elbow external fixator [6]. In all cases the distal humeral pin caused the lesion. The authors therefore recommended placing the pins in the distal humerus through an open approach. Similarly Livani and Belangero [5] and Apivatthakakul et al. [7] recommended an incision of at least 3–5 cm in length for minimally invasive plate osteosynthesis of the humerus. This facilitates blunt dissection and enables access to the bone under direct vision. Another measure to reduce the risk of iatrogenic injury to the radial nerve in the distal part of the humerus is supination of the forearm. It has been shown that supination moves the radial nerve more laterally and pronation more medially [7]. If the plate is placed on the anterolateral column in order to avoid the coronoid fossa, a Kocher approach may be safer than an anterior approach [5].

This case report does not put the minimally invasive plate osteosynthesis into question, but it demonstrates the importance of a correct surgical technique. Percutaneous fixation is associated with a high risk of iatrogenic damage to the radial nerve and is considered a surgical error. A sufficiently long skin incision and a blunt dissection of the soft tissues are mandatory to minimize this risk.
